# Weight control behaviors in overweight/obese U.S. adults with diagnosed hypertension and diabetes

**DOI:** 10.1186/1475-2840-8-13

**Published:** 2009-03-06

**Authors:** Guixiang Zhao, Earl S Ford, Chaoyang Li, Ali H Mokdad

**Affiliations:** 1 Division of Adult and Community Health, National Center for Chronic Disease Prevention and Health Promotion, Centers for Disease Control and Prevention, Atlanta, Georgia, USA; 2 Institute for Health Metrics and Evaluation, University of Washington, Seattle, Washington, USA

## Abstract

**Background:**

Obesity is a major risk factor for development and progression of hypertension and diabetes, which often coexist in obese patients. Losing weight by means of energy restriction and physical activity has been effective in preventing and managing these diseases. However, weight control behaviors among overweight/obese adults with these conditions are poorly understood.

**Methods:**

Using self-reported data from 143,386 overweight/obese participants (aged ≥ 18 years) in the 2003 Behavioral Risk Factor Surveillance System, we examined the proportion of overweight/obese adults who tried to lose weight and their weight control strategies by hypertension and/or diabetes status.

**Results:**

Among all participants, 58% of those with hypertension, 60% of those with diabetes, and 72% of those with both diseases tried to lose weight, significantly higher than the 50% of those with neither condition (Bonferroni corrected P < 0.017 for all comparisons). The multivariate-adjusted odds ratio (AOR) for trying to lose weight was 1.11 (95% confidence interval [CI]: 1.05–1.17) in participants with hypertension, 1.02 (95% CI: 0.90–1.15) in participants with diabetes, and 1.18 (95% CI: 1.07–1.29) in participants with both diseases (participants with neither condition as the referent). Among 78,446 participants who tried to lose weight, 23% of those with hypertension only and 28% of those with both hypertension and diabetes reported adopting a low fat/low calorie (LF/LC) diet in controlling their weight, significantly higher than 19% of those with neither disease (Bonferroni corrected P < 0.017 for all comparisons). Participants with both diseases had a significantly lower percentage of adopting physical activity in controlling their weight than those with neither condition (6% versus 12%, P < 0.01). After multivariate adjustment, the AOR for adopting a LF/LC diet plus physical activity to lose weight was 1.46 (95% CI: 1.15–1.84) in participants with both diseases. The AOR for adopting a LF/LC diet only to lose weight was 1.72 (95% CI: 1.35–2.20) in participants with both diseases and was 1.21 (95% CI: 1.03–1.40) in participants with hypertension only.

**Conclusion:**

The proportion of overweight/obese patients with diagnosed hypertension and/or diabetes who attempted to lose weight remains suboptimal and the weight control strategies varied significantly among these patients.

## Introduction

The rising trend in overweight and obesity has been a serious and growing public health problem in the United States [1-3]. From 1976–1980 to 2001–2004, the prevalence of overweight/obesity increased by 39% (from 47.4 to 66.0%) and the prevalence of obesity increased by 113% (from 15.1 to 32.1%) [1,2], the latter has increased slightly to 34.3% during 2005–2006 [4]. Obesity is associated with an increased risk of developing hypertension and diabetes [5-14]. In fact, the prevalence of diagnosed hypertension and diabetes has increased significantly from 1988–1994 to 2001–2004 (21.7% versus 26.7% for hypertension, 5.4% versus 7.3% for diabetes) [1]. In addition, the prevalence of obesity has doubled from 25.7% during 1976–1980 to 50.8% during 1999–2004 among people with hypertension [15]. Moreover, strong associations between a higher body mass index (BMI) and risk of hypertension or diabetes exist even among people within a normal BMI range [9,16].

A growing body of evidence has shown that losing weight by means of energy restriction and/or increasing physical activity has beneficial effects on the prevention and management of both diseases. A meta-analysis of randomized controlled trials on people with or without hypertension showed that an average weight loss of 5.1 kilograms reduced systolic blood pressure by 4.4 mm Hg and diastolic blood pressure by 3.6 mm Hg [17]. Among overweight/obese adults, increasing amount of intentional weight loss was associated with a linear decrease in diabetes incidence [10,18], and active weight loss is an effective approach to the treatment of people with diabetes [19-22]. Moreover, intentional weight loss is also associated with a significant reduction in all-cause mortality rate in people with or without diabetes [23-26].

Presently, little is known about weight control behaviors among overweight/obese adults with hypertension, diabetes, or both in the U.S. Given an increasing scientific and media attention on the multiple health benefits weight control/weight loss confers, we hypothesized that overweight/obese people with either hypertension or diabetes are more likely to attempt to lose weight (with the highest seen in people with both hypertension and diabetes) compared to people with neither condition. Using data from a nationally representative sample, we examined the proportion of overweight/obese people who attempted to lose weight and their weight control strategies among those with diagnosed hypertension, diabetes or both. We hope this study will increase our understanding concerning weight control behaviors in light of the increasing trends in overweight/obesity, hypertension, and diabetes in the U.S. population.

## Methods

Data for our analyses came from the Behavioral Risk Factor Surveillance System (BRFSS), a population-based telephone survey of health-related behaviors regarding the leading causes of death among noninstitutionalized U.S. adults aged ≥ 18 years. The BRFSS survey design, sampling methods and weights have been described elsewhere [27], and BRFSS data have consistently been found to provide valid and reliable estimates when compared to other national household surveys in the U.S. [27-29]. The survey was reviewed by the Human Research Protection Office at the Centers for Disease Control and Prevention and determined to be exempt from human subject guidelines. Further information on BRFSS is available at .

In 2003, a total of 149,324 participants who were overweight/obese (BMI ≥ 25 kg/m^2^, calculated from self-reported weight and height) were interviewed in all 50 states, the District of Columbia, and three U.S. territories of Guam, Puerto Rico and the Virgin Islands. The median cooperation rate (the percentage of eligible persons contacted who completed the interview) was 74.8%.

Respondents' hypertension and diabetes status were assessed by asking them whether they had ever been told by a doctor or other health professional that they had had these conditions. Those who answered that they had not been told they had hypertension (or diabetes) or they had these conditions only during pregnancy were categorized as "no diagnosed hypertension (or diabetes)". Respondents were then categorized as 1) having both hypertension and diabetes, 2) having hypertension only, 3) having diabetes only, and 4) having neither disease. Weight control status was assessed by asking respondents whether they were trying to lose weight. For those who responded with a "yes" to the question, they were further asked whether they were eating less fat and/or fewer calories (defined as a low fat/low calorie [LF/LC] diet) or participating in physical activity or exercise to lose weight. The receipt of doctors' advice on weight loss was assessed by asking respondents whether in the previous 12 months a doctor, nurse or other health professional had given them advice about their weight. Their responses were 1) yes, lose weight; 2) yes, gain weight; 3) yes, maintain current weight; 4) no advice; and 5) don't know/not sure. We treated the first category as "receipt of weight-loss advice" and combined the rest 4 categories into "receipt of no advice on weight loss".

The demographic variables in our analyses included respondents' age, sex, BMI, race/ethnicity (non-Hispanic white, non-Hispanic black, Hispanic, and others), education levels (< high school diploma, high school graduate, some college/technical school, and ≥ college graduate), marital status (married, divorced, never married, and others), and employment status (employed for wages, self-employed, unemployed, and retired). Current smokers were those participants who had smoked ≥ 100 cigarettes during their lifetime and were still smoking. Current non-smokers were those who either had smoked <100 cigarettes during their lifetime or had smoked ≥ 100 cigarettes in their entire life but stopped.

After excluding from the analytical sample participants who refused to answer, had missing responses to any questions, or responded "don't know/not sure" to any questions (except for the question on receiving weight-loss advice), a total of 143,386 participants were included in our analyses. The percentages of overweight/obese adults who attempted to lose weight and adopted a LF/LC diet and/or physical activity to lose weight by hypertension and/or diabetes status were weighted to the state populations and age-standardized to the 2000 U.S. population. A Bonferroni corrected P-value (labeled as P value only in the text) was used for multiple comparisons. Logistic regression analyses were conducted to assess the odds ratios for trying to lose weight and for adoption of a LF/LC diet and/or physical activity to lose weight among people with hypertension and/or diabetes using people with neither condition as the referent. We used SUDAAN software (release 9.0, Research Triangle Institute, Research Triangle Park, NC) to account for the multi-stage, disproportionate stratified sampling design.

## Results

**Figure 1:**
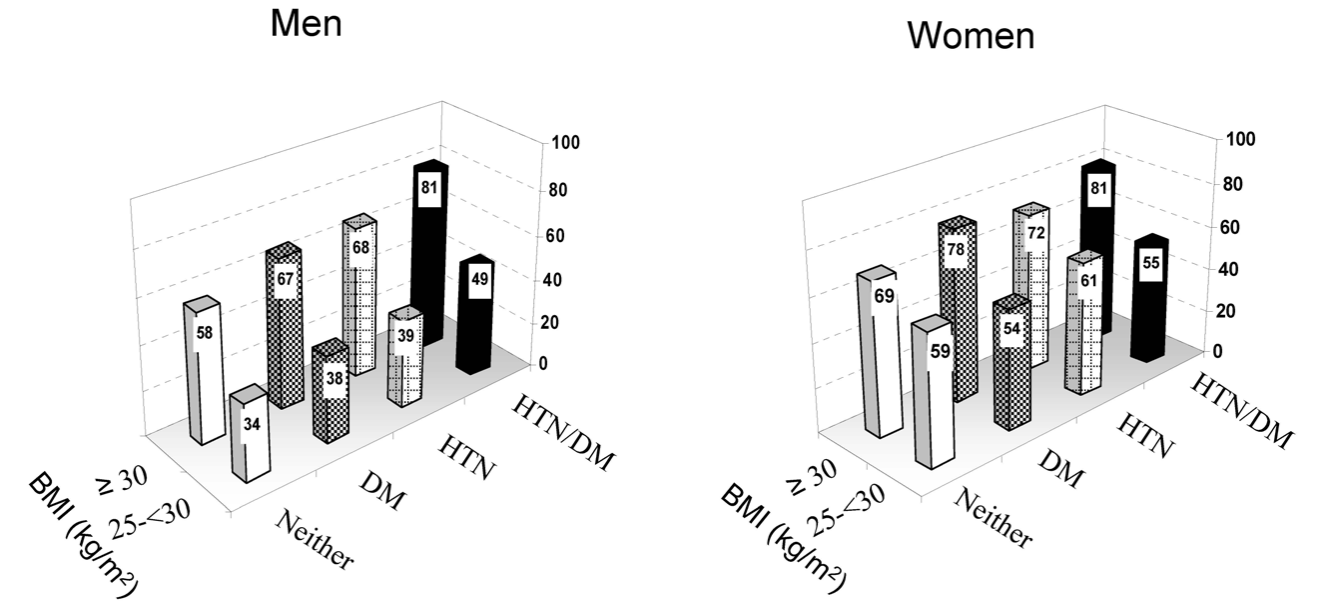
**Age-standardized percentages of men and women who attempted to lose weight by BMI and by hypertension and diabetes status, BRFSS, 2003**. HTN: hypertension; DM: diabetes.

Of all participants, 10,963 had both hypertension and diabetes, 40,666 had hypertension only, 5,143 had diabetes only, and 86,614 had neither condition. Overall, 72.1% (95% confidence interval [CI]: 68.7–75.2%) of those with both hypertension and diabetes, 57.8% (95% CI: 56.4–59.1%) of those with hypertension only, and 60.0% (95% CI: 56.2–63.8%) of those with diabetes only attempted to lose weight, significantly higher than the 49.8% (95% CI: 49.1–50.4%) of those with neither condition (P < 0.017 for all comparisons). The percentages of adults who tried to lose weight also varied significantly by gender and BMI levels (Figure 1 and Table 1). Overweight/obese women had a higher prevalence of trying to lose weight than overweight/obese men did except for those who were obese (BMI ≥ 30 kg/m^2^) and had both hypertension and diabetes concomitantly (i.e., 81% among obese women and men with both conditions). In addition, the prevalence of trying to lose weight increased with age till the age of 50–59 years and thereafter decreased; it was lower in non-Hispanic blacks than in non-Hispanic whites (P = 0.008), and was the lowest in those who were educated at less than a high-school diploma (P < 0.008) and who were current smokers among the selected categories (P < 0.017, Table 1). However, the prevalence of trying to lose weight was significantly higher in participants who received weight-loss advice than in those who did not (P < 0.001).

**Table 1:** Age-standardized percentages of overweight/obese adults who attempted to lose weight by selected characteristics, BRFSS 2003

**Characteristic**	**n**	**% (SE)**
**Total**	**143,386**	**52.8 (0.3)**
Disease status		
Having hypertension and diabetes	10,963	72.1 (1.7)
Having hypertension only	40,666	57.8 (0.7)
Having diabetes only	5,143	60.0 (1.9)
Having neither	86,614	49.8 (0.3)
Sex		
Men	67,734	44.5 (0.4)
Women	75,652	64.2 (0.4)
BMI (kg/m^2^)		
25.0-<30.0	87,200	44.3 (0.3)
≥ 30.0	56,186	66.6 (0.4)
Age (y)		
18–29	15,458	51.0 (0.8)
30–39	24,678	51.8 (0.6)
40–49	31,197	55.6 (0.6)
50–59	30,016	59.2 (0.5)
60–69	21,935	55.4 (0.6)
≥ 70	20,102	43.4 (0.7)
Race		
Non-Hispanic white	111,158	53.1 (0.3)
Non-Hispanic black	13,290	50.9 (0.8)
Hispanic	10,872	52.9 (1.0)
Other	8,066	51.7 (1.3)
Education		
< high school diploma	16,557	46.8 (0.9)
High school graduate	46,201	50.7 (0.5)
Some college/technique	39,301	55.4 (0.5)
≥ college graduate	41,327	55.5 (0.5)
Marital status		
Married	81,690	53.0 (0.4)
Divorced	20,482	51.8 (1.1)
Never married	19,091	53.9 (0.8)
Other	22,123	52.5 (0.9)
Employment		
Employed for wages	72,302	52.3 (0.5)
Self-employed	13,588	49.5 (1.0)
Unemployed	28,203	57.5 (0.6)
Retired	29,293	59.3 (5.3)
Smoking		
Current smoker	28,346	44.8 (0.6)
Former smoker	42,275	54.9 (0.6)
Never smoked	72,765	54.7 (0.4)
Receipt of weight loss advice		
Yes	29,972	80.6 (0.5)
No	113,414	46.0 (0.3)

BRFSS: Behavioral Risk Factor Surveillance System; SE: standard error

Among those who attempted to lose weight, 63.5% (95% CI: 62.8–64.2%) of them tried to lose weight by adopting a LF/LC diet and participating in physical activity, 21.0% (95% CI: 20.5–21.6%) by adopting a LF/LC diet only, and 10.9% (95% CI: 10.4–11.4%) by participating in physical activity only (Table 2). Overall, the percentages of adults who adopted a LF/LC diet plus physical activity did not differ significantly by hypertension/diabetes status; however, among those who were overweight, participants with both hypertension and diabetes had the highest prevalence (73.8%, 95% CI: 69.0–78.1%) of adopting a LF/LC diet plus physical activity to lose weight (P < 0.008). In addition, participants either with both hypertension and diabetes (27.6%, 95% CI: 24.4–31.1%) or with hypertension only (23.3%, 95% CI: 21.8–24.8%) had significantly higher percentages of adopting a LF/LC diet in controlling their weight, compared to those with neither condition (19.0%, 95% CI: 18.2–19.7%, P < 0.008 for both comparisons, Table 2). Among those who were overweight, participants with diabetes tended to have a higher prevalence of adopting a LF/LC diet only to lose weight; however, among those who were obese, participants with both hypertension and diabetes had a higher prevalence of adopting a LF/LC diet only than those with diabetes only (30.0% versus 22.0%, P < 0.008) or than those with neither condition (30.0% versus 23.0%, P < 0.008). Overall, participants with both hypertension and diabetes had a lower percentage of participating in physical activity to lose weight than those with hypertension only (6.3% versus 10.2%, P < 0.008) or than those with neither condition (6.3% versus 11.8%, P < 0.008).

**Table 2:** Age-standardized percentages of overweight/obese adults who attempted to lose weight by adopting a low fat/low calorie diet and/or engaging in physical activity, by BMI and hypertension/diabetes status, BRFSS 2003

	HTN/DM (n = 6,909)	HTN (n = 22,910)	DM (n = 3,015)	Neither (n = 45,612)	Total (n = 78,446)
	
	% (SE)	% (SE)	% (SE)	% (SE)	% (SE)
**Adopting a LF/LC diet and engaging in physical activity**					
BMI (kg/m^2^)					
25-<30	73.8 (2.3)	65.3 (1.2)	58.7 (4.9)	66.5 (0.6)	65.9 (0.5)
≥ 30	60.6 (2.4)	59.2 (1.2)	63.5 (2.7)	61.3 (0.7)	60.9 (0.5)
total	63.2 (2.0)	61.9 (0.9)	62.4 (2.4)	64.5 (0.5)	63.5 (0.4)
					
**Adopting a LF/LC diet only**					
BMI (kg/m^2^)					
25-<30	18.1 (2.0)	18.6 (0.8)	29.0 (5.1)	16.5 (0.4)	17.7 (0.4)
≥ 30	30.0 (2.0)	26.9 (1.1)	22.0 (1.8)	23.0 (0.7)	24.7 (0.4)
total	27.6 (1.7)	23.3 (0.8)	23.9 (2.1)	19.0 (0.4)	21.0 (0.3)
					
**Engaging in physical activity only**					
BMI (kg/m^2^)					
25-<30	5.0 (1.1)	11.7 (1.0)	7.7 (1.6)	12.8 (0.4)	12.2 (0.4)
≥ 30	6.5 (1.4)	9.0 (0.8)	9.5 (2.3)	10.4 (0.4)	9.5 (0.3)
total	6.3 (1.2)	10.2 (0.6)	9.0 (1.8)	11.8 (0.3)	10.9 (0.2)

BRFSS: Behavioral Risk Factor Surveillance System; SE: standard error; HTN/DM: having both hypertension (HTN) and diabetes (DM); LF/LC: low fat/low calorie

Compared to participants with neither disease, participants with either hypertension or diabetes or both were 2.3 to 3.9 times as likely to receive doctors' advice on weight loss (Table 3). However, after adjustment for socio-demographic variables and the receipt of weight-loss advice, only participants with both hypertension and diabetes or with hypertension only were significantly more likely to try to lose weight (Table 3). Among participants who attempted to lose weight, participants with both hypertension and diabetes were significantly more likely to lose weight by adopting a LF/LC diet plus physical activity or by adopting a LF/LC diet only after multivariate adjustment. In addition, participants with hypertension only were significantly more likely to adopt a LF/LC diet to control their weight (Table 3).

**Table 3:** Adjusted odds ratios (AORs) for trying to lose weight and receiving weight-loss advice in overweight/obese adults with hypertension and/or diabetes, or the AORs for adopting a low fat/low calorie diet and/or engaging in physical activity among those who attempted to lose weight (using people with neither disease as the referent), BRFSS 2003

	HTN/DM	HTN	DM	Neither
**Among overweight/obese people (n = 143,386)**				
Trying to lose weight†	1.18 (1.07–1.29)	1.11 (1.05–1.17)	1.02 (0.90–1.15)	1.00
Receipt of weight-loss advice‡	3.88 (3.52–4.29)	2.28 (2.14–2.44)	3.23 (2.81–3.71)	1.00
				
**Among overweight/obese people who attempted to lose weight (n = 78,446)**				
Adopting a LF/LC diet/engaging physical activity†	1.46 (1.15–1.84)	1.07 (0.92–1.23)	1.03 (0.77–1.37)	1.00
Adopting a LF/LC diet only†	1.72 (1.35–2.20)	1.21 (1.03–1.40)	1.05 (0.78–1.43)	1.00
Engaging in physical activity only†	0.96 (0.70–1.31)	0.95 (0.79–1.13)	0.80 (0.55–1.16)	1.00

†: Adjusted for age, sex, race/ethnicity, body mass index, education, marital status, employment, smoking, and receipt of weight-loss advice

‡: Adjusted for the same variables as listed above except for receipt of weight-loss advice.

BRFSS: Behavioral Risk Factor Surveillance System; HTN/DM: having both hypertension (HTN) and diabetes (DM); LF/LC: low fat/low calorie

## Discussion

Our results from a large, population-based survey sample showed that overall, only a little more than half of overweight/obese people tried to lose weight. Although overweight/obese people with hypertension only or with both hypertension and diabetes were 11 to 18% more likely to try to lose weight than those with neither condition, the proportion of these patients who tried to lose weight remains lower (ranged from 58 to 72%) than optimal. In addition, after multivariate adjustment, overweight/obese people with diabetes were not found to be more likely to lose weight than those without. Therefore, a gap remains wide even though the multiple health benefits of weight loss in control of hypertension and diabetes have been demonstrated.

To our knowledge, this is the first large study to examine weight control behaviors in overweight/obese people by hypertension and/or diabetes status. A few earlier studies reported that, during 1996–2000, 24% to 33% of men and 38% to 46% of women in the general U.S. population were trying to lose weight regardless of their BMI levels [30-32]. Among overweight/obese people, 48% of men and 66% of women were trying to lose weight [33]. We have previously reported that 59% of hypertensive women attempted to lose weight [34]. In the present study, we further demonstrated that only 58% to 72% of people with either hypertension or diabetes or both tried to lose weight. Moreover, contrary to expectations, we found that overweight/obese patients with diagnosed diabetes were only as likely as those with neither condition (hypertension and diabetes) to attempt to lose weight after multivariate adjustment for socio-demographic characteristics, smoking status and receipt of doctors' advice on weight loss, suggesting intensive intervention or education programs are needed for diabetes patients.

Weight loss by various strategies significantly reduces body fat mass, blood pressure, fast glucose and hemoglobulin A1c, and improves the 2-h glucose tolerance test, beta-cell function, and insulin sensitivity [17,19-22,35-37]. Adequate weight loss can also reduce the requirement (number and doses) of antihypertensive medications or anti-diabetic medications in patients with hypertension and/or diabetes [19-21,35,36,38]. In addition, weight loss produces a favorable lipid profile such as lowering total – and LDL-cholesterol, apolipoprotein B and triglycerides, and increasing HDL-cholesterol level [19,36,37,39], thereby decreasing cardiovascular disease risk in these patients. Thus, weight management is a key strategy in controlling hypertension and diabetes and their complications. It has been shown that behavioral counseling from physicians or other health care providers plays an important role in promoting weight loss in overweigh/obese patients [40-44]. Increased dialogue between diabetes patients and health care providers on behavioral goals significantly increased levels of physical activity and weight loss [40]. In the present study, although overweight/obese patients with either hypertension or diabetes or both were 2 to 4 times as likely as those with neither condition to receive doctors' advice on weight loss, these patients were only 2% to18% more likely to try to lose weight after further adjustment for this variable. This may result from some barriers for weight-loss counseling such as physicians' insufficient confidence, knowledge, and skill on weight management strategies [42]. Moreover, although several studies showed that the proportion of people trying to lose weight increased with increasing level of education in the general population [30-32], which is consistent with the findings of the present study, a study conducted by Gurka et al. reported that patients' educational background differentially affected the lifestyle intervention on weight loss in diabetes control [45]. Diabetes patients with lower educational levels (no college degree) who participated in lifestyle intervention lost more weight or waist circumference than those with higher levels of education (≥ college degree) [45]. In contrast, among usual care participants, patients with less education gained more weight or waist circumference than those with greater education [45]. This suggests that future weight-loss intervention programs or physicians' counseling on weight loss should be individualized in patients with diabetes in order to achieve behavioral goals.

A variety of weight-loss strategies have been implemented in the U.S. population including eating less fat or fewer calories, increasing physical activity or exercise, skipping meals, eating food supplements, taking diet pills, and taking water pills or diuretics [46]. Among those trying to lose weight, reducing fat/calorie intake was the most common strategy [31,33]. In the present study, two common strategies for weight control – eating less fat/fewer calories and increasing exercise/physical activity were examined. An encouraging finding of our study is that over 60% of overweight/obese patients attempted to lose weight by the combined strategies regardless of their disease status. Patients with both hypertension and diabetes were 46% more likely to lose weight by the combined strategies and 72% more likely to lose weight by eating a low fat/low calorie diet only than those with neither condition. In addition, patients with hypertension were 21% more likely than those with neither condition to lose weight by consuming a low fat/low calorie diet. However, our results indicate that greater weight control efforts are needed among overweight/obese patients with diabetes since, at the national level, we found these patients were not more likely to lose weight, and even in those who attempted to lose weight, they were neither more likely to eat a low fat/low calorie diet nor more likely to engage in physical activity. It is possible that overweight/obese people with diabetes only are not in severe condition so they may think weight management is not imperative in controlling their diabetes. However, given the great benefits of weight loss on improved glucose tolerance and insulin sensitivity in people with pre- or newly diagnosed diabetes [37,47], it is important to educate and encourage these patients to lose excess weight.

Our study has several limitations. First, all measures including weight control behaviors, disease status and BMI were self-reported, thus subject to recall bias. Second, our analyses were based on people with diagnosed hypertension and diabetes; therefore, the prevalence of trying to lose weight in undiagnosed hypertension and diabetes remains unknown. Moreover, as mentioned previously, the less frequent weight-loss strategies such as eating food supplements, taking diet or water pills, or taking diuretics were not evaluated by disease status because of lack of information on these weight control strategies. Those who reported trying to lose weight but neither adopted a low fat/low calorie diet nor engaged in physical activity may have used these strategies. Finally, five years have passed since the data for this analysis were collected (from the 2003 BRFSS). Abid et al. has reported that the proportion of obese persons who reported being counseled by a healthcare professional to lose weight was in a declined trend during the period of 1994–2000 [48]. Thus, updated weight control behaviors should be continuingly monitored at local, state, and national levels.

In summary, the proportion of overweight/obese patients with diagnosed hypertension and/or diabetes who tried to lose weight remains suboptimal and the weight control strategies varied significantly among these patients. Presently, attempting to lose weight is a common health behavior in overweight/obese people; it should be emphasized further in those with various obesity-related chronic comorbidities given the great health benefits of weight loss in controlling these diseases. Our results indicate that great efforts are needed from healthcare providers to interact with their patients to set suitable behavioral goals for weight loss and to provide appropriate education to promote effective weight-loss strategies in these patients. In addition, population-based obesity interventions to promote healthy eating, physical activity and energy balance would benefit all including patients with hypertension and/or diabetes.

## Abbreviations

AOR: adjusted odds ratio; BMI: body mass index; BRFSS: Behavioral Risk Factor Surveillance System; CI: confidence interval; DM: diabetes; HTN: hypertension; LF/LC: low fat/low calorie.

## Competing interests

The authors declare that they have no competing interests.

## Authors' contributions

GZ conducted the data analyses, interpreted the data and prepared the manuscript. ESF supervised the data analyses and contributed to the manuscript writing. CL and AHM made critical revisions of the manuscript for important intellectual content. All authors have read and approved the final version of the manuscript.

## Disclaimer

The findings and conclusions in this article are those of the authors and do not necessarily represent the official position of the Centers for Disease Control and Prevention.
